# Supplementary material for: Coupling of individual quantum emitters to channel plasmons
Source: Nat Commun. 2015 Aug 7;6:7883. doi: 10.1038/ncomms8883 (PMC4918332; doi:10.1038/ncomms8883)
Supplement: Supplementary Information — Supplementary Figures 1-6, Supplementary Table 1 and Supplementary Discussion [file ncomms8883-s1.pdf]

Supplementary Figures

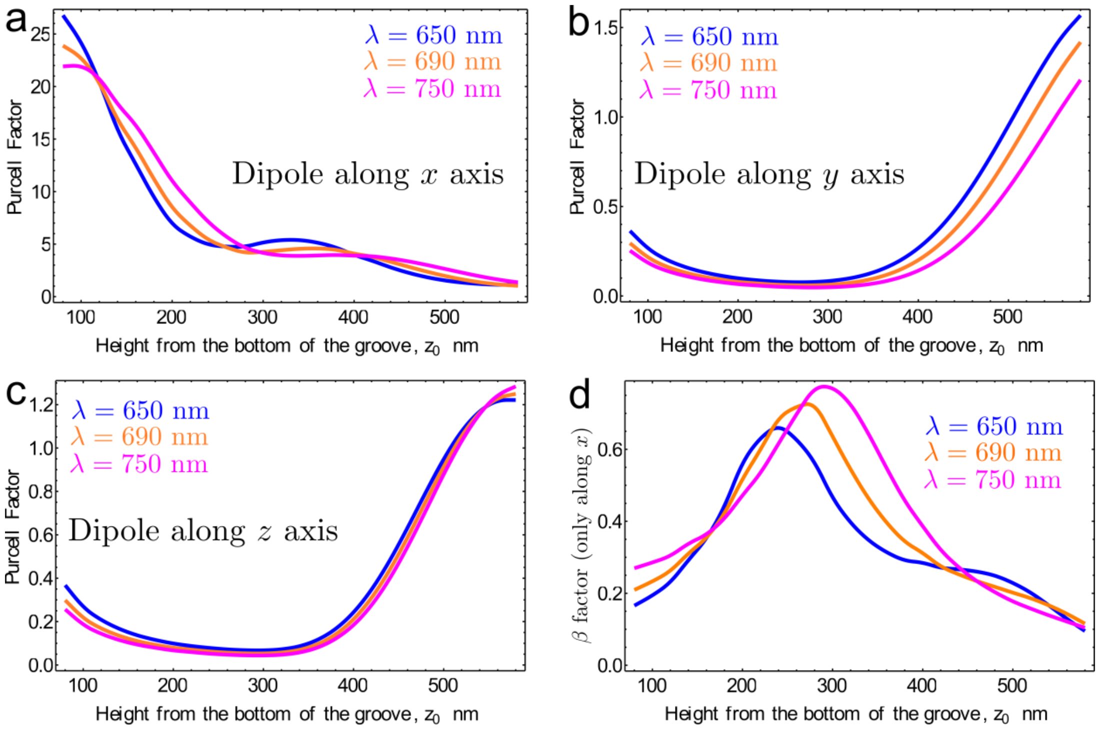

Supplementary Figure 1. **Purcell and beta factor without the diamond host for three wavelengths within the NV spectrum.** Purcell factor for a dipole oriented along the a) x-axis, b) y-axis and c) z-axis, and d) Beta factor for the orientation along the x-axis. For the y- and z-axis the beta factor was close to 0.

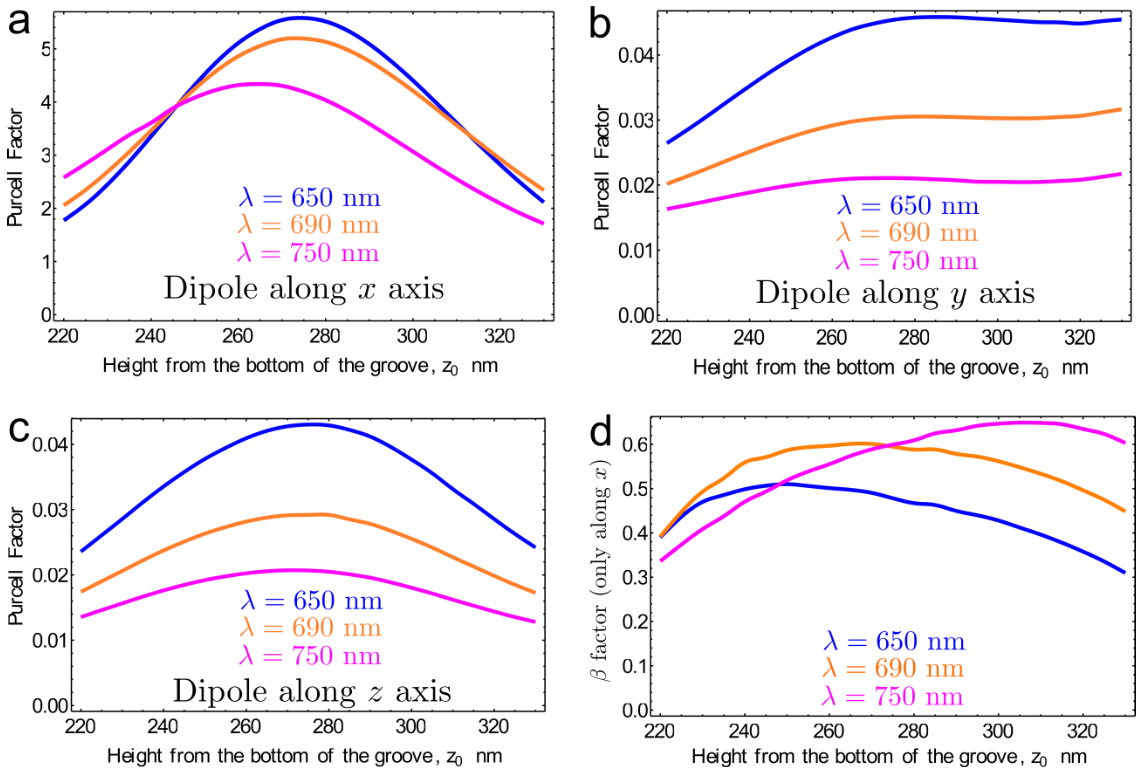

Supplementary Figure 2. **Purcell and beta factor with the diamond host for three wavelengths within the NV spectrum.** Purcell factor for a dipole oriented along the a) x-axis, b) y-axis and c) z-axis, and d) Beta factor for the orientation along the x-axis. For the y and z axis the beta factor was close to 0.

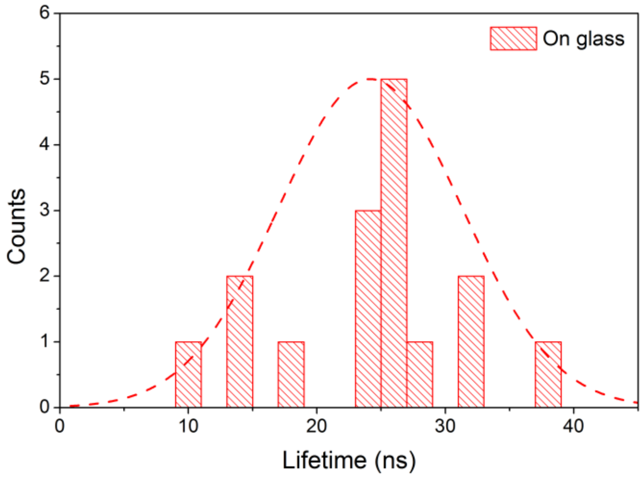

Supplementary Figure 3. **Lifetime distribution of NDs with single NV centres on a glass substrate.**

A total of 16 single NV-NDs were measured on a glass substrate yielding a lifetime distribution of 24.2 ± 7.2 ns.

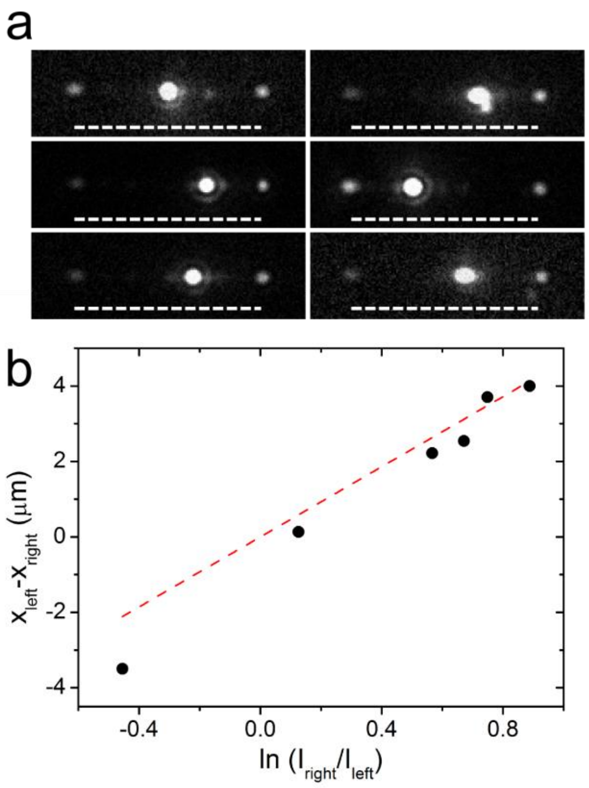

Supplementary Figure 4. **Propagation length estimation.** a) Wide field fluorescence EMCCD images from NDs containing multiple NV centres coupled to the CPPs of the VGs. The dashed scale bar is 10 μm. b) Experimental data extracted from a), with the red dashed curve a linear fit to equation (2). We obtain a propagation length value of 4.65 um ± 0.48 μm.

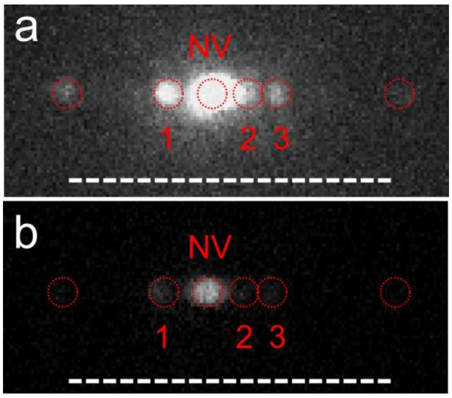

Supplementary Figure 5. **Coupling of a single NV centre deterministically positioned into a VG plasmonic waveguide.** a) Saturated and b) unsaturated wide field fluorescence EMCCD images of the NV-VG device without a polarizer in the collection channel. The excitation polarization is set parallel to the VG. The dashed scale bar is 10 μm.

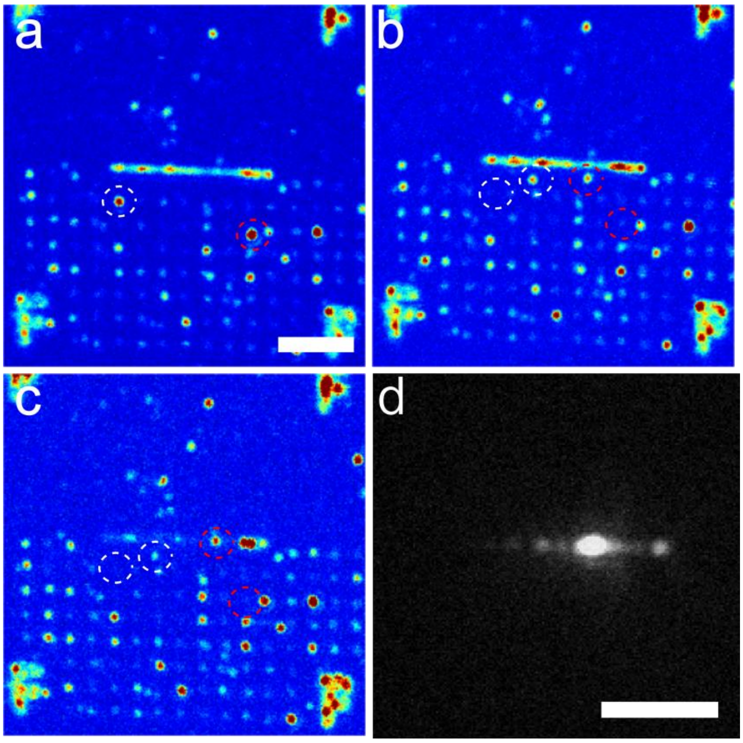

Supplementary Figure 6. **Assembly and coupling of device #2.** a-c) Confocal scans demonstrating the deterministic positioning of two selected nanodiamonds (white and red circles). In the 2nd step (c) only the particle labelled in red circle was positioned inside the VG. d) Saturated EMCCD image of the NV-VG device evidencing the coupling into the CPP modes by the NV centre. Scale bars are 5 μm.

Supplementary Table

	Central spot	Left VG end	Right VG end	Outcoupler1	Outcoupler2	Outcoupler3
Distance to central spot (μm)	0	4.9	6	1.6	0.9	1.9
Mean value	2389368	93348	43503	468898	247660	242239
Std.Dev	25808	4068	2287	15940	67917	15734

Propagation Length	4.6
Std. Dev.	0.5

Beta factor	0.42
Std. Dev.	0.03

Supplementary Table 1. **Beta factor estimation based on the intensity counts and accounting for the intrinsic propagation losses of the VG.**

Supplementary Discussion

Propagation length. To extract an approximate propagation length value of our VGs we performed measurements on a sample with nominally equivalent VGs and to which we randomly deposited nanodiamonds with multiple NV centres per particle (~15 NVs). As the assembly is random, we obtained NDs placed at different positions along the VG. The Supplementary Figure 4 shows a set of wide field fluorescent EMCCD images of different ND-VG devices, evidencing the out-coupling of the CPPs at the nanomirror positions. From these images we can extract the ratio of intensities between the two out-coupling spots and the relative distance of the nanomirrors to the central excitation spot. In this approach we assume that the intensity of the CPPs propagating along the VG is described by the following exponential function:

$$I = I_o e^{-\alpha x} \tag{1}$$

where I is the measured intensity of the emission at the nanomirror position, I_o is the emission coupled into the CPP mode by the emitters (in one direction), $\alpha = 1/L_{\mathrm{pl}}$ with L_{pl} the propagation length of the CPP mode supported by the VG, and x is the distance between the nanomirror and the ND. We assume the coupling to be symmetrical to both forward and backward directions, the losses to be uniform along the waveguide and the out-coupling efficiency of both nanomirrors to be equivalent. From this equation we can derive a simple expression to relate the intensities and relative distances as follows:

$$x_{\mathrm{l}} - x_{\mathrm{r}} = L_{\mathrm{pl}} \ln\left(\frac{I_{\mathrm{r}}}{I_{\mathrm{l}}}\right) \tag{2}$$

with the subscripts referring to the intensities and distances for the left and right out-coupling spots with respect to the central confocal spot. Values are extracted from the images in the Supplementary Fig. 4 and fitting them with equation (2) leads to a propagation length value of 4.65 um ± 0.48 μm. This value is in good agreement with the simulations of the intrinsic propagation length, where we extracted a weighted value of 4.56 μm when considering three wavelengths (650 nm, 690 nm and 750 nm) and their contributions within the NV spectrum.

Coupling efficiency. To estimate the coupling efficiency of the NV centre emission into the CPP modes of the VG some assumptions must be made. First, we consider that the non-radiative rate has a negligible contribution in our hybrid device based on the fact that we work with rather large nanodiamond particles, so that in the case of a dipole emitter at the centre of the VG, the distance of the QE to the metallic walls of the VG is of at least 50 nm and thus in a regime where non-radiative rates are not dominant over radiative and plasmon related decay channels. We consider the collection efficiency from different positions along the VG to be uniform as all of the emission is coupling out from a diffraction limited channel. The intensity values from each out-coupling spot are corrected to account for the experimental propagation loss extracted in the previous section. Under these assumptions the coupling efficiency is then estimated by the following relation:

$$\beta = \frac{I_\mathrm{l}e^{\alpha x_\mathrm{l}}+I_\mathrm{r}e^{\alpha x_\mathrm{r}}+I_1e^{\alpha x_1}+I_2e^{\alpha x_2}+I_3e^{\alpha x_3}}{I_{NV}+I_\mathrm{l}e^{\alpha x_\mathrm{l}}+I_\mathrm{r}e^{\alpha x_\mathrm{r}}+I_1e^{\alpha x_1}+I_2e^{\alpha x_2}+I_3e^{\alpha x_3}} \quad (3)$$

where $\alpha = 1/L_\mathrm{pl}$, as in equation (1), and the subscripts for the intensities and the x values correspond to those labelled in Supplementary Fig. 5. The Supplementary Table 1 summarizes the corresponding measurements and results extracted from the unsaturated EMCCD image presented in Supplementary Figure 4. For this device we obtain a coupling efficiency of 0.42 ± 0.03 with an associated Purcell factor of 2.3 ± 0.7.

Additionally in the Supplementary Figure 5 we present a similar device assembled with the same procedure as the device presented in the main text, from (a)-(c) one can see how two selected nanodiamonds where brought close to the VG and eventually only one of them positioned into the VG. The coupling to the CPP modes supported by the VG is evidenced in (d). For this device we estimate a coupling efficiency of 0.41 ± 0.05 with an associated Purcell Factor of 2.1 ± 0.6.
